# Supplementary material for: Experiences of COVID-19 infection in North Carolina: A qualitative analysis
Source: PLoS One. 2022 Jun 2;17(6):e0269338. doi: 10.1371/journal.pone.0269338 (PMC9162358; doi:10.1371/journal.pone.0269338)
Supplement: S1 File — (DOCX) [file pone.0269338.s001.docx]

**English Interview Guide**

**Introduction to the interview**

This interview is structured in two parts. First, we will first ask you about your personal experiences with COVID-19. This part will begin with the time before you were diagnosed up and will continue through the time when you recovered and were ready to resume your regular activities, if you have reached that point. Second, we would like to get your opinion and suggestions on general ideas about how COVID-19 influenced you and your community’s daily lives.

There are no right or wrong answers and we greatly appreciate any input on the topic you want to provide to us.

Do you have any questions before we get started?

**Experience of COVID-19 infection**

Please tell me a little bit about yourself including:

How old are you?

Where you live and with whom?

Where do you work?

How do you usually get around?

How do you spend the majority of your time?

Pre-diagnosis: Before you started feeling symptoms or were tested:

1. What did you know about COVID-19 and how it spreads?
2. From where did you usually get your information about COVID-19?
3. What did you think about your personal risk of getting COVID? Why?
4. Tell me about any specific actions you took to reduce your risk of getting infected.
5. Were there any specific actions that you wanted to take in order to reduce your risk of getting infected but were unable to?

Diagnosis: Thank you for speaking with me so openly. I am interested in learning about your journey from when you were first diagnosed with COVD-19 until you recovered and how you experienced that time.

1. When, in days to weeks before your first test, did you first think you might have gotten COVID-19? This can be due to your own symptoms, or being in close contact with someone who was diagnosed.
2. What happened that made you decide to get a test?
3. How long did it take you to decide to get a test?
4. How and where did you get tested?
5. How long did it take to get a test after you decided to get one, and how long did it take for the results?
6. Overall, how did you feel about the process of being tested?
7. How did you feel about the care you received when being tested?
8. Once you got the diagnosis, what was on your mind? What did you think about?
9. After you have gotten the test results to what extent, if any, were you able to stay isolated?
10. How did this impact you, your family, or your community?

**Treatment/recovery course**

1. At what point did you decide to go see a doctor?

2. How did you decide where to get care for your illness during this process?

3. How did you get there?

4. What happened then?

*Only for those hospitalized*

5. Did you initially want to stay at home or be in the hospital? Why?

6. How did you feel about your care and the care team?

7. What could have been improved?

*Both groups*

8. What were your interactions like with other people like your friends, family, and community while you had COVID-19?

9. How did your infection impact them?

10. Did you have any concerns or worries regarding your family and community during the time you were sick? [Examples to provide if needed by subject: ability to protect others, ability to isolate, ability to secure food and income, coping]

11. What do you think would have made that time easier for you?

12. What, if any, community resources or social service organizations did you use to help you or your family while you were sick? Were there any others that you are aware of or wanted to use but couldn’t? Why do you think that was the case?

**After recovery:**

1. How are you feeling now?

2. How has getting COVID impacted your life? [Topics to include if subject needs additional information: Day to day, Mental/behaviorally, Physically, Financially]

3. What kind of resources or support do you need after your COVID-19 infection that you did not need before?

**General influences on COVID experience**

We are now at the end of the interview and I would like to summarize and learn more about your general thoughts on the COVID-19 pandemic, if there are any parts of this that we did not talk about before.

1. How has the COVID-19 pandemic, even before you became infected, impacted your personal life?

2. In your own opinion, what were the biggest factors that influenced your own experience with COVID-19? [Factors to consider if subject needs examples: Health insurance status, transportation, poverty, ability to isolate/housing, employment, family support, community support, local government responses, policies and relief efforts]

3. What was missing in the overall response throughout this pandemic?

4. Why do you think so?

Conclusion

We have now come to the end of the questions that I had for you. I thank you very much for being so open and honest with me. Do you have any additional questions, or is there something you would like to add? [Have I missed something that you would like to talk about]?

Thank you.
