# Supplementary material for: Experiences of COVID-19 infection in North Carolina: A qualitative analysis
Source: PLoS One. 2022 Jun 2;17(6):e0269338. doi: 10.1371/journal.pone.0269338 (PMC9162358; doi:10.1371/journal.pone.0269338)
Supplement: Attachment — Submitted filename: Response to Reviewers.docx [file pone.0269338.s002.docx]

**Response to Reviewers**

**Experiences of COVID-19 Infection in North Carolina: A Qualitative Analysis**

*We thank the Reviewers for their careful critiques and constructive suggestions. Below we provide a point-by-point response to reviewer comments. We have used “track changes” in a marked-up copy to indicate changes made in the manuscript. Below we have added page numbers where Reviewers may find relevant text/changes when in the unmarked version of the manuscript.*

**Journal Requirements:**

Comment: Please ensure that your manuscript meets PLOS ONE's style requirements, including those for

file naming.

Response: We greatly appreciate the notice, and we have revised the manuscript accordingly. Additionally, we have uploaded the Supplement S1 file with a copy of the qualitative interview script as described in the manuscript, which was not included in the previous submission.

Comment: We note that the grant information you provided in the ‘Funding Information’ and ‘Financial Disclosure’ sections do not match.  When you resubmit, please ensure that you provide the correct grant numbers for the awards you received for your study in the ‘Funding Information’ section.

Response: Thank you, we have entered the correct information for the Emergency Medicine Foundation grant that supported this work in the “Funding information” section, and it should now match the original “Financial Disclosure” section. The EMF does not use grant numbers for their awards. However, we note an error in the "Financial Disclosure" section, as one of the authors was not included in the list of Associate investigators. It should read "JS, AT, AL, CS, JRNV" instead of "JS, AT, CS, JRNV". We were not able to find the option to edit this directly in the resubmission, thank you.

**Review Comments to the Author:**

Comment: Reviewer #1: Although qualitative study can potentially provide insight into this critical COVID pandemic issue, I would love to see more details coming from higher sample number and fair comparison between different groups. This manuscript has low impact.

Response: We thank the reviewer for their comment, and appreciate that this is a critical COVID pandemic issue. Our intention with this study was to focus collectively on patients living in high risk COVID-19 hotspot communities as a group that would be high-yield targets for public health interventions and could additionally inform larger studies in the future. We did not intentionally sample from different groups for the purposes of comparison, but this would be an excellent direction for future work, and we thank the reviewer for this idea. We believe that we yielded rich responses from our sample size with 5 predominant themes and 17 sub-themes discussed in this paper, and that we achieved data saturation. This is consistent with prior work finding that 6-7 interviews will capture the majority of data using inductive analytic methods, and that 11-12 interviews is consistent with the very highest levels of saturation [1]. We have added a statement to the methods section on “Interview Procedure/data collection” on page 8 to address this. Please additionally see the response to the reviewer comment on a stopping rule for data collection below as it additionally informs our sample number.

Comment: Reviewer #2: This study reports the results of a qualitative interview study of ten North Carolinians who were diagnosed with COVID-19 in the first three waves of the pandemic. The results of the interviews provide new information about individual beliefs during the pandemic and reactions to the health care system both before (e.g. testing), during, and after (long-covid) their illness.

Reviewer: We greatly appreciate these kind comments recognizing the novel data in this manuscript.

Comment: Major items - I appreciate that the focus of this study is qualitative, however, it isn’t clear if the conclusions the authors draw from the data are supported by the data since it is unclear how many people are endorsing each of the themes/sub-themes the authors identify. It would be helpful if table 2 was augmented with the number of interviewees who mentioned each sub-theme.

Response: We thank the reviewer for this excellent suggestion. We have added numbers throughout the text of the manuscript to establish the numbers of patients who discussed each of the themes and subthemes included. We elected to use the text of the manuscript as opposed to the table, as it allows for more detail in each subtheme, but we believe that this addresses the reviewer comment.

Survey issues

Comment: What was the response rate?

Response: We appreciate the opportunity to clarify this point. 158 eligible participants were identified in the hotspot clusters, and using available contact information in their health records, 10 patients agreed to participate. We have included a statement with this in the methods section on “Interview Procedure/Data Collection” on page 7.

Comment: Which “census area” did the authors use to identify hot spots?

Response: Thank you for this question, we used the census tract level. We have changed the language in the methods section on “Setting, Participants, and Recruitment” on page 6 to reflect this.

Comment: The sample is very different from the population of North Carolina. Can the authors provide a comparison with the demographic characteristics of the identified hot spots?

Response: Thank you for the chance to clarify this point. Our randomized sample was taken equally from each of the census tract level cluster of White, Black, and Hispanic populations, as our intention was to collect data from patients of all groups living in hotspot communities, although this would not correspond to the overall demographics of North Carolina. We acknowledge that our patient sample (self-identified as 7 Black, 3 White, and 1 Hispanic Mexican) is not equally representative of all these groups, and future studies could specifically highlight each group. We have added a statement to the limitations section on page 31 to acknowledge this.

Comment: What was the stopping rule for data collection?

Response: We appreciate this helpful question to strengthen this manuscript. Our stopping rule was based on reaching information saturation, which we defined as when few or no new concepts emerged in subsequent data collection through our inductive content analysis approach. At this point, we reached a nuanced understanding of the perspectives of participants living in our defined COVID-19 hotspot communities. We have added a statement to clarify this in the methods section on “Interview procedure/data collection” on page 8.

1.	Guest G, Namey E, Chen M. A simple method to assess and report thematic saturation in qualitative research. PLoS One. 2020;15(5):e0232076.
